# Putting the individual in the context of the organization: A Carnegie perspective on decision-making

**DOI:** 10.3389/fpsyg.2023.1165713

**Published:** 2023-10-27

**Authors:** Daniel A. Levinthal, Daniel A. Newark

**Affiliations:** ^1^Wharton School, University of Pennsylvania, Philadelphia, PA, United States; ^2^HEC Paris, Jouy-en-Josas, France

**Keywords:** organizational decision-making, judgement and decision-making, behavioral theory of the firm, organizational learning, decision-making in context

## Abstract

The majority of decision research portrays decision-makers as largely decontextualized, separate from the institutional and social factors that influence their choosing. On the occasions when context is considered, it is rarely organizational, despite the prominence of organizations in people's lives. By contrast, the Carnegie perspective on decision-making emphasizes context, particularly that of organizations, as a central concern. We develop this contrast by first reviewing the limited role of context in neoclassical economic and psychological depictions of choice. Next, we present key elements of the organizational decision context in the Carnegie perspective: decision premises, standard operating procedures and decision rules, organizational structures, learning environments, and identity–situation interaction. We then consider the importance of interpretation to decision-making in context. In particular, rather than being given and clear, the meaning of decision context is often ambiguous and must be interpreted or constructed. The Carnegie perspective underscores the importance of this interpretive process to both decision-making and everyday life. We conclude by considering aspects of context that merit greater examination, as well as the implications for behavioral theorizing of acknowledging the contextualized nature of action.

*No man is an island*,

*Entire of itself;*

*Every man is a piece of the continent*,

*A part of the main*.

–Donne (1624/1987)

## Introduction

John Donne's admonition to recall our connectedness notwithstanding, many of the core traditions within behavioral decision theory seem to have largely decontextualized action, judgment, and decision-making. In this sense, much of the scholarship on choice can be subject to Granovetter's (1985) broad critique of economic accounts of individuals and firms as under-embedded or under-socialized. Despite this general property of research on choice, we suggest that the Carnegie perspective has long been mindful of the importance of context to choice, while still preserving a fundamental belief in individual agency. For the Carnegie perspective, the primary context is organizations. Simon (1948/1997) set out to understand administrative behavior, judgement, decision-making, and action in a hierarchical structure. Cyert and March (1963/1992) sought to understand and develop a parsimonious representation of firm decision-making. March (2010) and March and Olsen (1984, 1989, 1995, 2006) extended the notion of context to include broader social and cultural norms and values, as filtered through organizational life, as well as the need for decision-makers to interpret the context within which they operate.

We first put forward some of the important lines of decision-making research that we suggest serve as counterpoints to the Carnegie perspective, beginning with the classic conceptions of rational choice in the economics literature, followed by Kahneman and Tversky's alternative formulation of behavioral decision theory, the elaboration of choice architecture introduced by Thaler and colleagues (Thaler and Sunstein, 2008; Benartzi et al., 2017), and the research program of Gigerenzer and colleagues on ecological rationality (Gigerenzer and Gaissmaier, 2011; Todd and Gigerenzer, 2012). With these building blocks in place, we shift to a discussion of how the Carnegie perspective presents a contrasting, more richly contextualized view of choice. We consider some of the key ways in which an organization serves as a context that influences choice through decision premises (Simon, 1948/1997), standard operating procedures and decision rules (March and Simon, 1958; Cyert and March, 1963/1992), organizational structures (Dearborn and Simon, 1958; Cyert and March, 1963/1992; Cohen et al., 1972; Ocasio, 1997), learning environments (Levitt and March, 1988; Haunschild and Miner, 1997; Argote and Miron-Spektor, 2011), and the interaction between decision-makers' identities and the situations in which they find themselves (March, 1994; March and Olsen, 2006).

Furthermore, acknowledging the importance of context underscores the importance of interpretation, often in the face of ambiguity (March, 2010). Early work in the Carnegie tradition highlighted aspiration-based, dichotomous encoding of experience as either “success” or “failure” (March and Simon, 1958; Cyert and March, 1963/1992). However, subsequent work treats experience as having far greater ambiguity and latitude for interpretation than the success vs. failure dichotomy of aspiration learning suggests (March, 2010; Newark, 2014; Levinthal and Rerup, 2021). Individuals tell stories to themselves and others, creating narratives to give meaning to, and create understanding of, their lives and experiences. While such narratives can be over-determined, causal explanations are a powerful mechanism for sense-making in a complex and otherwise confusing world. These acts of interpretation are not only fundamental to decision-making but, as work from the Carnegie perspective has regularly suggested (Cyert and March, 1963/1992; March and Olsen, 1975, 1984; Feldman and March, 1981; March and Sevón, 1984; March, 1987, 1994, 1999, 2010; Newark, 2014, 2018, 2020), may be fundamental to our efforts to create meaning and understanding in our lives more broadly.

In sum, the Carnegie perspective gives us a conceptual apparatus with which to consider organizationally situated action. As Gavetti et al. (2007, p. 528) noted, “Any conception of an organization that omits a notion of individuals who are situated in distinct places in some structural arrangement will be hard pressed to engage much of what we commonly experience in organizational life… The original conception of organizations in the Carnegie School did in fact provide such a theoretical apparatus.” We aim to bring forth this theoretical apparatus, both by contrasting alternative conceptions of decision-making and by highlighting the richness of contextualizing factors emphasized by the Carnegie perspective.

## Neoclassical economics: context as markets

Both for work in psychology (Kahneman and Tversky, 1979) and for work within the Carnegie perspective (Simon, 1955), the conception of choice and action provided by neoclassical economics was a referent and touchstone in efforts to articulate a behaviorally grounded view. The neoclassical approach reduces decision-making to the mathematical operator of maximization: that is, maximization of a utility function for individuals and of profits for firms. Individuals may differ in the information available to them and in their preferences, but they are homogenous in their impeccable, consistent, Spock-like judgment processes. In that sense, the characterization of decision-making is divorced from the context of actual individual capabilities and vicissitudes.

However, context does enter the neo-classical framework in an important respect. While utility functions and firm profits are not observable, Samuelson (1947), in a key contribution of his *Foundations of Economic Analysis*, introduced the idea of comparative statics, which showed how the constrained optimization approach to economics that he developed could make empirical predictions even in the absence of knowledge of the objective function (utility or profit) to be maximized. Changes in relative prices (of goods and services for consumers, of capital and labor for firms) lead to predictable, qualitative (directional) changes in consumers' consumption choice as well as a firm's production technology. In this sense, context is critical to the neoclassical apparatus, as empirical predictions from the model only stem from changes in the “context” in which actors operate. Further, context is also present not only in market forces (in the form of prices of inputs and outputs), but also in focal others, especially in game-theoretic treatments of competitive interaction (Von Neumann and Morgenstern, 1944).

However, these “contexts” are external—encompassing market prices and other firms—and not internal with respect to the firm itself, as is central to the Carnegie perspective. Even agency theory (Jensen and Meckling, 1976), which slightly opened up the convenient “black box” of firms as owner-operator entities relied on by neoclassical economics, remains a theory of contractual relationships. Although there may be important distinctions between contractual relationships among actors within an enterprise and those external to it (Baker et al., 2002), the ultimate conception, as Holmstrom (1999) terms it, is the firm as a “subeconomy” of economic relationships.

## Behavioral decision theory

### Heuristics and biases: context as framing

Kahneman and Tversky posed a stark challenge to the neoclassical paradigm. While the rational choice framework imposes no restrictions on what might constitute individual preferences, and in particular individuals' risk preferences, it does impose a constraint of consistency. Kahneman and Tversky showed that when facing choice problems with mathematically equivalent probabilistic payoffs, individuals vary their choice as a function of how the problem is framed or represented. These anomalies served as a behavioral puzzle and a starting point for their theorizing. Their aim, as manifest in their work on prospect theory, was to take the expected utility apparatus of neoclassical theory and modify it into a theory of choice that was consistent with the experimental data on the judgment tasks they and others had examined. In this regard, Kahneman and Tversky (1979) offered two key modifications of the expected utility apparatus. One was the characterization of an individual's perceived value over payoffs, postulating that individuals are risk averse in the domain of gains (a concave payoff function) and risk-seeking in the domain of losses (a convex function).^1^ The other key modification was to introduce a subjective probability construct, with the property that small objective probabilities close to zero would be over-weighted and large objective probabilities close to one would be under-weighted. With these two modifications of the expected utility apparatus, Kahneman and Tversky were able to reconcile various anomalies, such as the Allais Paradox.

Clearly this was path-breaking work and helped set the course for a rich line of inquiry on behavioral decision theory. Context was critical in this framework in the sense of choice attributes or other aspects of what Thaler and his collaborators (Thaler and Sunstein, 2008; Benartzi and Thaler, 2013; Thaler et al., 2014; Benartzi et al., 2017) termed choice architecture (e.g., whether outcomes are framed as gains or losses, the order in which alternatives are presented, the presence of anchors, the setting of defaults, the presence of irrelevant alternatives that may nonetheless influence preferences). However, these were nonetheless experiments and theorizing around individual judgments, with decision-makers still largely removed from any kind of social or institutional context, such as other people, relationships, roles, places, or history. Decision-makers were depicted as islands of judgment. Thus, while the work on choice architecture brings to the fore the scaffolding surrounding judgment tasks, that scaffolding is not a social context, but rather quite specific features of the decision frame. Even more recent research on judgment and decision-making that has begun to pay greater attention to social context has tended to ignore the particular context of organizations in favor of broader cultures and norms (e.g., Miller, 1999; Weber and Morris, 2010; Yates and De Oliveira, 2016)—a practice consistent with social psychological research more generally (Lewin, 1951; Tajfel, 1972; Gergen, 1973; Markus and Kitayama, 1991; Heath and Sitkin, 2001; Staw, 2016), and one that underscores the uniqueness of Carnegie's organizational focus.

### Ecological rationality: context as the familiar environment

Gigerenzer and his collaborators (Gigerenzer and Brighton, 2009; Gigerenzer and Gaissmaier, 2011; Todd and Gigerenzer, 2012) critique the work on prospect theory and the broader judgment and decision-making program for focusing excessively on the potential downsides of decision biases and heuristics, while neglecting their potential efficacy and efficiency. In doing so, they call for investigating “ecological rationality,” which takes into account an individual's environment when assessing the desirability of a particular heuristic. Context clearly plays an important role in this line of research, generally in the form of an actor's familiarity with their environment or choice. For instance, it is possible to predict with 91% accuracy which of two cities in Germany has a larger population based on which city has a team in the Bundesliga, the German professional soccer league (Gigerenzer et al., 1991). Thus, when one city has a professional soccer team and one does not, presence of a professional soccer team is a good heuristic for predicting relative city size. In this way, the individual is no longer making choices in a “vacuum,” but in a world in which they have lived and with which they have some familiarity. It is not a world, however, of organizational processes or even social interactions. This holds even when the choice context has organizational attributes, such as when an experienced manager may learn to classify a customer as active or inactive using the heuristic of how many months it has been since the customer's last purchase (Wübben and Wangenheim, 2008), or when a manager may make better hiring decisions using a heuristic rather than logistic regression (Luan et al., 2019). Further, the tasks, or empirical tests, are generally limited to ones of prediction or knowledge of basic facts, with clear right answers. They are not considerations of collective action and decision-making and, in that regard, fail to capture much of the dynamics of choice in organizations or other social contexts.

## The Carnegie perspective: context as organizations

### Decision premises

As Simon (1991) notes, while much of the discourse in economics focuses on the role of markets, most economic activity occurs within firms. Indeed, the prominent role of business organizations is a key characteristic of developed economies. For pre-industrial societies, families and clans were the primary social structure. In modern industrial (or post-industrial) societies, the business organization has become and remains a focal social structure in individuals' lives. From its beginnings with Simon's *Administrative Behavior* (Simon, 1948/1997), the focus of the Carnegie perspective has been on behaviorally grounded accounts of decision-making in the context of organizations.

A central mechanism by which organizational context impacts individual action in Simon's (1948/1997) argument is decision premises. Higher-order actors within an organization provide what the contemporary literature would term logics (Thornton and Ocasio, 2008; Thornton et al., 2012) by which those working under their guidance and authority should act. These premises, or logics, do not delineate specific actions or decisions, but rather a basic framework within which those actions should be taken or choices made. There are some links to the work on choice architecture noted previously, but choice architecture generally references a rather specific and narrow element of a decision's framing, such as whether saving for retirement or donating one's organs is something one opts into or out of when filling out a form (Johnson and Goldstein, 2003; Benartzi and Thaler, 2013). In contrast, a decision premise serves as a less specific but far more robust mechanism by which choices are influenced. For instance, a decision premise might guide a product development team at a technology firm by providing a sense of the firm's values, such as the design aesthetic of Apple, the commitment to high technical performance of BMW, or the importance of sustainability at Patagonia. In this sense, decision premises help actors navigate the inevitable trade-offs that they face.

The notion of decision premises as developed in Simon's (1948/1997) early work suggests a hierarchical cascade from higher-order premises down to more specific goals across different facets of the organization. However, just as March (1962) problematizes the possibility of a superordinate goal for an organization, as one moves from more abstract, higher-order premises to the more concrete, lower-order goals intended to align with and effectuate them, these more granular lower-level goals may end up at odds with each other. Thus, while decision premises, like goals, are intended to help direct individual action and coordinate collective behavior (Greve, 2023), both mechanisms are subject to the possibility of inconsistencies and potentially conflicting implications. In our discussion of organizational structures below, we examine some of the key lines of argument within the Carnegie perspective on the quasi-resolution of goal conflict and the possibility of effective collective action despite its presence.

### Standard operating procedures and decision rules

March and Simon (1958) and Cyert and March (1963/1992) shift from an emphasis on decision premises to the role of standard operating procedures and decision rules. This shift is particularly pronounced with Cyert and March—and was likely prompted in part by the desire to specify a computational model of organizational decision-making. *A Behavioral Theory of the Firm* presented a conceptual framework by which one could understand both a boundedly rational, goal-directed entity subject to some degree of internal conflict and corresponding computational models of such a structure. Decision-making in the model operates in the form of a series of “if–then” rules. If a certain condition holds, then a certain action is taken. In parallel to this effort, Newell and Simon (1972) pioneered early efforts in artificial intelligence in which domain experts presented with a choice situation would engage in protocol analysis as they were asked to verbalize the logic by which they made their judgements. These “protocols” were then codified computationally (Figenbaum, 1978).

Decision premises and decision rules are both mechanisms by which the social structure in which individuals operate—i.e., the organizational context—influences and guides action. They do not eliminate individual agency or reduce decision-makers to Tayloristic automatons. Discretion remains. But the decision calculus, as influenced by decision premises and standard operating rules, is deeply impacted. Further, while writings in the Carnegie perspective tend not to speak extensively of organizational culture and norms, decision premises and rules can be seen as mechanisms by which such factors influence decision-making and action.

Related to the idea of decision premises is the notion of a firm's strategic context: a line of argument introduced in the works of Simon and Cyert and March, and later elaborated by Bower (1970). Bower pointed to the role of the firm's strategic context in inducing action by managers, making it a mechanism by which managerial initiatives could be directed. Burgelman (1983) applied these ideas to corporate entrepreneurship and later developed the arguments further, highlighting the dual role of autonomous initiatives (not guided by the strategy context) and induced initiatives (guided by this context) in understanding the dynamics of strategic change (Burgelman, 1991). Levinthal's (2017, 2021) development of the firm as an “artificial selection” environment and the role of a “Mendelian executive” in molding this environment is a further elaboration of these ideas, and an effort to link the Carnegie perspective with that of evolutionary economics (Nelson and Winter, 1982).

### Organizational structures

Adam Smith (1776) famously highlighted the division of labor as an engine of the development of individual expertise and overall economic progress. Interestingly, and perhaps an under-appreciated point, his argument for the division of labor did not hinge on some form of arbitrage or gains from trade, as later introduced by Ricardo (1817). Rather, Smith emphasized that specialization in a task would generate skill in that specialized function—a pin-maker was not born with a particular skill in one of the eighteen steps that Smith identifies in this, his motivating example, but rather became skilled through hours of experience in the task.

Task structures play a central role in the Carnegie perspective's conception of decision-making situated in organizations, as there is a close connection between the division of labor and the division, or specialization, of cognition (Dearborn and Simon, 1958; Cyert and March, 1963/1992). Similar to Smith's argument, Dearborn and Simon (1958) demonstrate how given roles and tasks influence actors' cognitive schema. The marketing manager, the production engineer, and the CFO see the world through qualitatively different lenses. In a similar vein, Chase and Simon (1973) demonstrate the different ways novices and experts interpret a common stimulus—in their case, the position of chess pieces on a game board. In particular, experts are able to “chunk” the data in ways that allow them to more efficiently encode the information, while novices interpret the data as individual pieces on the board.^2^

Divergent roles not only create divergent cognitive schema, they also create potentially divergent goals (Cyert and March, 1963/1992). March (1962) addresses this challenge of organizational goals from the vantage point of what the contemporary management literature would term “stakeholders,” considering the possibly divergent interests of front-line workers, management, investors, and others. In doing so, he illuminates the stark behavioral differences between an individual with a goal (a depiction common across decision-making research) and an individual with a goal in an organization populated by others with conflicting goals (a depiction relatively unique to the Carnegie perspective).

March begins with an argument in the spirit of Arrow's (1951) work on social choice and his “impossibility theorem” regarding the challenge of aggregating individual preferences into a coherent choice structure (i.e., one involving a transitive preference ranking). However, while Arrow stops with his impossibility result, March ventures further and poses the possibility of coalitions of actors and the associated possibility of coalition power and politics reconciling these divergent interests. Per the role of power, the viability of a coalition does not imply that all parties' interests are treated equally, but rather that all parties receive sufficient payoff relative to their outside options such that they are willing to participate in the coalition.

Cyert and March (1963/1992) point to facets of organizational processes and structures as contextual mechanisms that allow the organization to achieve some over-arching sense of direction and coordinated action in the face of these divergent interests. In particular, they note the potential power of sequential allocation of attention amongst otherwise non-reconcilable goals. For instance, an organization may seek both growth and efficiency. Addressing these two imperatives simultaneously may present challenging trade-offs and choices for managers. Alternatively, focusing first on growth, with efficiency as more a constraint than a goal, provides a higher degree of clarity of action and decision-making. Then subsequently, the organization might come to believe that it would be better served by shifting its focus toward efficiency. Neither objective is deemed intrinsically more important than the other; rather, each has its day.

A structural, rather than temporal, mechanism for contending with competing interests is having one organizational unit focus on a particular objective while another unit focuses on a different objective. Thus, there is not a unifying high-order goal, but rather there are local, unit-specific objectives. The degree to which a solution proves effective depends on how modular, or nearly decomposable, the task structures are across the organizational units (Ethiraj and Levinthal, 2004, 2009). If the pursuit by subunit A of its sub-goal materially impacts the payoffs to subunit B in terms of its own distinct sub-goal, then a specialized goal structure can lead to dysfunctional perturbance of one unit's problem-solving effort by another's (Ethiraj and Levinthal, 2004). While this work points to the importance of modularity, and essentially horizontal task division, Levinthal and Workiewicz (2018) note that a key aspect of Simon's (1962) notion of nearly decomposable systems was a vertical dimension of structure and the possibility of actions at a lower level being encapsulated in some summary fashion. Organizations as structures of decision-making, building on early work by Sah and Stiglitz (1986), has served as an important line of work that links two key pillars of the Carnegie perspective: decision-making and task structures (Knudsen and Levinthal, 2007; Christensen and Knudsen, 2010; Csaszar, 2012).

In their work on the “Garbage Can Model,” Cohen et al. (1972) point to the possibility of more complex organization structures that allow for a more fluid mapping of actors, and even issues, to choice opportunities. While the Garbage Can Model is often associated with organized anarchy and the relatively free flow of people, problems, and solutions, the formal model itself makes no assumption about whether the flows are highly structured or lacking in structure. The model provides an analytical framework within which to consider how alternative structures influence these flows and the implications for organizational decision-making—including the potential absence of decision-making (i.e., decision by oversight or flight in the model). Ocasio (1997) builds on these ideas and develops what he terms an attention-based view of strategy. This line of work (Ocasio, 1997; Ocasio and Joseph, 2005; Joseph and Ocasio, 2012) explores how organizational structures and organizational restructuring impact the strategic decision-making process by making distinct agendas, markets, and actors more or less salient.

### Learning in context

Organizational learning has been a central theme in the Carnegie tradition, and one intimately intertwined with choice (March and Olsen, 1975). The basic engine of learning processes is reinforcement—actions associated with positive reward are reinforced and those associated with negative outcomes tend to be avoided (Thorndike, 1932). Context is a central, though not always highlighted, feature of such learning processes. For instance, what one learns about being an effective employee with one manager may not translate well to another manager, let alone to a different organization. This role of context bears keeping in mind as management scholars increasingly turn to randomized controlled trials (RCTs) as a mechanism of learning. RCTs avoid the possible misleading implications one might draw from “natural” samples in which individuals and firms select into specific treatment conditions, creating problems of causal inference. However, as Levinthal (2021) observes, such studies often under-attend to the context-dependence of their findings by failing to consider the representativeness or generalizability of their samples. An RCT is itself a context-dependent mechanism of learning.

Levinthal and March (1993) point to important biases in feedback-driven processes, stemming from their tendency to be myopic. Feedback that is more proximate in time and space within the organization is likely to be more salient. Cost-cutting measures in a focal unit give a clear and immediate signal of progress. Developing novel processes and products offers more distant possible returns that may not necessarily benefit the innovating unit. As a result, they argue that learning processes will tend to privilege exploitation over exploration.

A critical part of the learning context is the broader ecology of learning processes within which any learning occurs (Levitt and March, 1988). Levinthal and March (1981) introduce the pathology of competency traps, stemming from the fact that people in organizations are simultaneously learning what actions to take as well as developing efficacy in the actions they have chosen. As a result of this dual learning process, organizational actors may view as unattractive policies, initiatives, and technologies with which they are inexperienced, despite the latent superiority of these alternatives. Levinthal's (1997) work on fitness landscapes provides another mechanism for competence traps, stemming from the interdependencies of actions. As a result of these interdependencies, shifting an initiative of a single actor may seem unattractive even if shifting a broader set of initiatives could lead the organization to a superior peak in the landscape. Local search in a setting of high interdependence will tend to lead organizations to local, rather than global, peaks.

Argote and Miron-Spektor (2011) highlight additional ways in which features of the organization impact learning processes. They note that “a context where members share a superordinate identity has been found to lead to greater knowledge transfer (Kane et al., 2005). Similarly, contexts where members trust each other (Levin and Cross, 2004) or feel psychologically safe (Edmondson, 1999) have been found to promote organizational learning” (Argote and Miron-Spektor, 2011, p. 1125).

Another key contextual source of learning is other organizations (Levitt and March, 1988; Haunschild and Miner, 1997). Of course, other organizations can provide misleading lessons as well as useful insights. Diffusion processes may be driven by a practice's merit, but they may also be driven by processes of legitimacy and status (Haveman, 1993). Moreover, given the interdependence of practices and processes, what may be a useful practice in one context may prove less useful in another. Benchmarking, comparing the efficacy of practices across a set of organizations, assumes either highly homogenous entities or limited interdependencies—a world of universal “best practices” (Levinthal, 2021). Going beyond the specific confines of the Carnegie perspective, organizational sociology points to the role of network structures in influencing the knowledge of practices and behaviors across organizational populations (Burt, 1980; Davis and Greve, 1997; Beckman and Haunschild, 2002; Reagans and McEvily, 2003).

### The logic of appropriateness: context as identity, situation, and their interaction

Organizations serve not only as structural arrangements, but also as bases of identity (Ashforth et al., 2011; Ashforth and Schinoff, 2016). To say that one works at Google or ExxonMobil both conveys information to others and influences one's image of oneself. Values and norms are ascribed to organizations, and those values and norms can both be attributed to individual members and also serve as templates for these members. Of course, even our professional identities are not wholly circumscribed by the organizations we are part of or our particular role in them. We have a variety of roles and identities, both professional and personal (Ramarajan, 2014), that originate beyond the organization's boundaries. For instance, professions—e.g., medicine, law, architecture—provide individuals with a set of norms and values quite apart from those of the particular organizations to which they belong. Moreover, one's values may stem in part from a commitment to a particular community, set of religious beliefs, or other broader social norms beyond one's professional life.

As work on the logic of appropriateness (March and Olsen, 1989, 1995, 2006; March, 1994; Messick, 1999; Weber et al., 2004; Newark and Becker, 2021) emphasizes, those identities may be central to understanding the behavior and motivations of individuals in organizations. This work makes an important distinction between the logic of appropriateness and the logic of consequences. The logic of consequences underlies the reasoning of intended rationality, such as expected value calculations and cost–benefit calculations. By contrast, the logic of appropriateness attends less to the desirability of potential outcomes and more to the accordance of actions and behaviors with one's identity or role, given the situation one is in. This means that the fundamental question of the logic of appropriateness is not “Which decision alternative has the most desirable or sufficiently desirable expected consequences?” but rather, “What does a person such as I do in a situation such as this?” (March, 1994). The context of both one's organizational identity or role and the organizational situation in which one finds oneself are paramount.

For instance, the logic of appropriateness suggests that an on-duty soldier would follow an order from their commanding officer not because of some calculation of the costs and benefits of adherence vs. disobedience, but because that is what an on-duty soldier does in that situation. The Hippocratic Oath and its modern variants, taken by new doctors around the world, are not business plans regarding healthcare reimbursements, but a series of commitments to what constitutes appropriate actions for a medical professional in situations of care. As work in organizational theory and psychology (Messick, 1999; Weber et al., 2004; March and Olsen, 2006; Newark and Becker, 2021) has noted, logics of appropriateness bring organizational context to the fore, calling for actors to consider what kind of person they are, what sort of situation they are in, and what such a situation demands of such a person.

### Interpretation as input: giving meaning to context in order to make decisions

Thus far, we have explored various manifestations of context in the Carnegie perspective that have an important influence on individual choice behavior. But exactly what that influence is will depend not only on one's context in some objective sense, but also on one's subjective interpretation of that context. The Carnegie perspective has emphasized the construction of meaning from its earliest days and, particularly with the post-1970s writings of March (1982/2005, 1987, 1994, 1999, 2010; see also March and Olsen, 1975, 1984; Feldman and March, 1981; March and Sevón, 1984; and Cyert and March, 1963/1992), the consideration of these processes grew increasingly rich. The key insight is that the meaning of the decision context is neither given nor unambiguous, but rather must be constructed and is often subject to multiple interpretations.

In early writings within the Carnegie tradition, the question of “meaning” was largely confined to the basic question of whether an observed outcome (e.g., a sales figure or profits) should be categorized as success or failure, with the aspiration level (March and Simon, 1958; Cyert and March, 1963/1992) demarcating these two domains. The dynamics of aspiration levels and their possible implications for organizational search have, as Gavetti et al. (2007) suggest, arguably been the most developed element of the behavioral theory of the firm (cf., Greve, 2003).

However, subsequent interest in the interpretation of context has gone well beyond this categorization of outcomes as successes or failures. To begin, all components of choice have to be interpreted and imbued with meaning. As March (1999, p. 25–26) put it,

Theories of rational action assume that decision makers make sense of their situation by forming expectations about future consequences and preferences for those consequences. Theories of rule-based action assume that decision makers make sense of their situation by identifying situations as matching identities and rules and by interpreting the implications of those matches. Decisions are seen as predicated on these meanings that are established prior to action.

In addition to interpreting information to arrive at expected consequences and expected preferences for those consequences (in the case of a logic of consequences) and interpreting a multiplicity of potential identities, situations, and proper behaviors (in the case of a logic of appropriateness), decision-makers must also interpret experience. As March and Olsen (1975, p. 148) noted, “organizations adapt their behavior in terms of their experience, but that experience requires interpretation. People in organizations come to believe what happened, why it happened, and whether it was good; but the process by which those beliefs are established in the face of a quite problematic 'objective' world affects systematically what is learned.”

A key technology for this interpretation of context is talk, particularly in the form of stories or narratives (March et al., 1991; March, 2010; Newark, 2014). Storytelling is fundamental to how context is not only interpreted and learned from, but also constituted. This makes understanding how stories are constructed and which stories are most likely to be adopted central to understanding how context shapes choice and how situated decision-making unfolds.

### Interpretation as output: making decisions in order to give meaning to context

As March and Sevón (1984, p. 102) noted, “Perhaps interpretation is more a primary feature of human behavior than a servant of choice. From such a perspective, information is sought and considered because it contributes to understanding what is going on in life; and understanding what is going on is important independent of any purpose to which the knowledge might be put.” The ephemerality of existence may tilt the balance of what is important away from achieving desirable decision outcomes and toward the interpretations of life we construct and the stories we share while we make decisions (March and Olsen, 1984; March, 1994; Newark, 2014, 2018, 2020, 2021). In this way, interpretation is seen less as an instrumental activity that facilities choosing (i.e., an input into the choice process) and more as an end in itself (i.e., an output of the choice process) (Feldman and March, 1981; March, 1999; Levinthal and Rerup, 2021).

This is a view that sees decision-making processes not primarily as a means for achieving desirable decision outcomes, but rather as an occasion, excuse, or catalyst for interpreting life. Choices provide an arena to contemplate and constitute our context, and in the end contemplating and constituting context may be as or more important than the alternatives we select or the outcomes in which those alternatives result. This view led March (1999, p. 28) to suggest that,

“Decision making may, in many ways, be better conceived as a meaning factory than as an action factory. Decision outcomes are often not as central to an understanding of decision making as might be expected. Individuals and organizations write history and construct socially acceptable story lines about links between actions and consequences, identities and behaviors. Decision making is a prime arena for developing and enjoying an interpretation of life and one's position in it.”

## Conclusion

The 17^th^ century French philosopher René Descartes famously said, “I think therefore I am.” It is a deductive argument that cogitation is what constitutes and defines. However, as suggested by our opening reference to Donne's poem, individuals are not isolated and do not cogitate in vacuums. Individuals are part of institutional and social contexts, with organizations being one of the most central. And so, while Descartes proposed “I think therefore I am,” Don Quixote, the protagonist of Miguel de Cervantes' classic eponymous novel, guides and justifies his behavior by asserting, “I know who I am” (March, 2011). To know who one is, one must understand where one is; one must understand the context in which one is situated.

While Quixote poses the contextualization of thought and action in a rather grand manner, this issue operates in more prosaic ways in our daily lives in organizations. A marketing manager knows what to do because their role and the task environment in which they operate provide strong guidance, and their years of experience in that role, in the focal organization and others, provide a lens and set of constructs that inform understanding. The Carnegie perspective brings the “Spock-like” creation of neoclassical economics and the isolated decision-maker of psychology to context—a context of organizations populated by rules and routines, challenged by conflicting goals and power dynamics, and animated by values and identities, all of which require interpretation.

Recognizing the importance of context poses challenges for us in our role as social scientists and as social engineers. As scientists we seek understanding, ideally understanding that might be couched in terms of general “laws” or insights. Nevertheless, trade-offs between generalizability, accuracy, and parsimony are omnipresent. This means that, as social scientists, we are generally relegated to what Merton (1949) termed mid-range theories or March (2008, p. 5–9; see also Liu et al., 2015), even more modestly termed “little ideas”: ideas that “provide propositions about a small set of phenomena within a small set of contexts” and that “identify and explore small mechanisms of limited scope capable of producing notable effects and possibly susceptible to empirical verification.” From this perspective, “context” operates as a kind of rate-limiting property on social science progress and our capacity to give advice.

For many scholars, incorporating context often means making fairly elaborate contingency arguments. However, being mindful of context need not lead to contingent truths. Instead, one can offer specific insights, like that exploitation tends to drive out exploration, or general challenges, like the ambiguity of experience. These insights, while profound, are also partial; they do not form a single, grand unifying theory. As a result, there are reasons to be cautious regarding the application of such insights to a particular organization in its specific circumstances. Practices in a given organization might be foolish and ripe for improvement. However, they may also represent contextualized wisdom and the embodiment of situated experience. Organizational designers, consultants, and social architects should be informed and guided by scholarship's “little ideas,” but modest about knowing their implications in any particular setting at any particular time, and appreciative of the possible wisdom of current practices. As March (2006, p. 84) noted, “If a manager asks an academic consultant what to do and that consultants answers, then the consultant should be fired. No academic has the experience to know the context of a managerial problem well enough to give specific advice about a specific situation.”

While the Carnegie perspective has done much to bring context into our consideration of decision-making, and thereby has supplemented more decontextualized economic and psychological accounts, it could be useful to revisit Carnegie's micro-foundations. The psychology literature has made enormous strides since Simon in the mid-1950s sought to create a behaviorally grounded counterpoint to neoclassical economics. Work within the Carnegie tradition could benefit from an infusion of these contemporary insights from psychology. For instance, the conception of action has been largely devoid of the role of emotion. In that sense, the Carnegie perspective moved away from the Spock-like character of neoclassical economics, but offered instead something of a “Tin Man” sensibility of an actor without a heart. Better accounting for the role of affect (Barsade et al., 2003; Loewenstein and Lerner, 2003) in organizationally contextualized decision-making would provide a richer depiction of choice.

Scholars operating within the Carnegie perspective should also be leery of the possible competence traps of path-dependent learning, not letting origins in Simon's conception of bounded rationality prove overly deterministic. Indeed, one of the defining characteristics of the Carnegie perspective is operating as an open and living line of inquiry in which ideas and insights might diffuse and evolve (March, 2005; Gavetti et al., 2007, 2012; Beckman, 2021), rather than a more narrow, calcified “school of thought” with rules for what constitutes legitimate interpretation of the associated ideas and membership in the “school.” At the same time, the organizations literature, and essential features of the Carnegie perspective in particular, enriches our understanding of decision-making, whose processes often do not occur on isolated islands of autonomous individuals, but rather in the context of social institutions and organizations.

## Author contributions

All authors listed have made a substantial, direct, and intellectual contribution to the work and approved it for publication.

## Footnotes

^1^While having some superficial similarity to the notion of aspiration levels in the Carnegie tradition, the two frameworks are quite distinct. Aspirations are a mechanism by which outcomes are encoded into a discrete category of success or failure. This is an ex-post mechanism—having experienced an outcome, how does an individual view it? Prospect theory looks ex-ante at the choices individuals face and how their view of potential gains and losses stemming from those choices influence those choices. Thus, while both frameworks entail a reference point, it is important to bear in mind the ex-post learning, interpretation focus of aspirations and the ex-ante risk–return assessment of prospect theory.

^2^Chase and Simon (1973) present an important and illuminating contingency. Experts have an advantage when pieces follow standard patterns of moves and counter-moves, but their advantage is eliminated if the pieces are arrayed at random. This contrast makes clear that it is not a difference in raw memory at work, but rather a difference in representations and schema.
